# Antidepressant- and Anxiolytic-like Effects in Mice of Alkaloids from Aerial Parts of *Argemone platyceras* Link & Otto

**DOI:** 10.3390/ph18010049

**Published:** 2025-01-03

**Authors:** Mayra Beatriz Gómez-Patiño, Rosa Estrada-Reyes, Héctor Hugo Hernández-Mendoza, Ángela Suarez-Rojas, Daniel Arrieta-Baez

**Affiliations:** 1 Instituto Politécnico Nacional, Centro de Nanociencias y Micro y Nanotecnologías, Unidad Profesional Adolfo López Mateos, Av. Luis Enrique Erro S/N, Colonia Zacatenco, Mexico City 07738, Mexico; mbgomez@ipn.mx; 2 Laboratorio de Fitofarmacología, Dirección de Investigaciones en Neurociencias, Instituto Nacional de Psiquiatría Ramón de la Fuente Muñiz, Calzada México-Xochimilco 101, Col. San Lorenzo Huipulco, Tlalpan, Mexico City 14370, Mexico; restrada@inprf.gob.mx; 3 Laboratorio de Productos Naturales y Síntesis Orgánica, Facultad de Ciencias Básicas, Ingeniería y Tecnología, Universidad Autónoma de Tlaxcala, Calzada de Apizaquito S/N, San Luis Apizaquito, Tlaxcala, Apizaco 90401, Mexico; hectorhugo.hernandez.m@uatx.mx (H.H.H.-M.); angela.suarez.r@uatx.mx (Á.S.-R.)

**Keywords:** *Argemone platyceras*, antidepressant effect, anxiolytic effect, direct infusion electro-spray (DIESI), UHPLC-MS

## Abstract

**Background/Objectives:** *Argemone platyceras* Link & Otto, an endemic plant of Mexico, is widely distributed in the central area of the country, mainly in the states of Tlaxcala, Puebla, and the State of Mexico. Ethnobotanical studies in different communities of these states have demonstrated that it is primarily used to treat diabetes and mental illnesses, such as “los nervios” (nerves) and “el ansia” (anxiety); these terms are used in traditional medicine, but it is accepted that they refer to anxiety disorders. This study aimed to validate the traditional use of aerial parts of *A. platyceras* Link & Otto in treating these illnesses. **Methods:** a standardized acidic method to obtain alkaloids was used to obtain an extract (AlkExt), which was tested in adult male Swiss Webster mice in the tail suspension (TST) and forced swimming (FST) tests. **Results:** AlkExt was analyzed using mass spectrometry techniques (DI-ESI and UHPLC-MS) to detect 2,3′,4,5′-Tetramethoxystilbene (*m*/*z* 301.14, 3%), scoulerine (*m*/*z* 328.16, 19.8%), tetrahydro-columbamine (*m*/*z* 342.17, 28.8%), 8-(hydroxymethyl)-2,10-dimethoxy-6,8,13,13a-tetrahydro-5H-isoquinolino[2,1-b]isoquinoline-1,11-diol (*m*/*z* 358.17, 22.8%), and glaucine (*m*/*z* 356.19, 11.1%); these were assayed in a single oral administration of AlkExt, which caused robust anxiolytic- and antidepressant-like effects without affecting the spontaneous ambulatory activity of the mice. **Conclusions:** The easy and standardized AlkExt analyzed in pharmaceuticals assays in this study strongly suggest its therapeutic potential to treat the comorbidity of anxiety and depression disorders and support further investigations in people with these diseases.

## 1. Introduction

Depression and anxiety are highly prevalent mood disorders and health problems that affect more than 300 million people worldwide. The World Health Organization has indicated that it is the leading cause of disability worldwide [1]. Even though there are effective drugs for depression, medicinal plants are an important therapeutic alternative and the primary source of health for many communities in the world. Therefore, the search for new molecules to treat these disorders is ongoing.

The use and systematization of plants in traditional Mexican medicine, based on the experience and practices of indigenous cultures, has been passed from generation to generation and has played an essential role in its current use in the prevention and treatment of various diseases [2]. Within this context, in the country’s central region, mainly in Puebla, Tlaxcala, Hidalgo, and the State of Mexico, various plants have been used to treat neurological diseases, including depression and anxiety [3,4].

Through multiple scientific studies, the empirical use of medicinal plants to treat mental illnesses such as “los nervios” (nervous) has been documented [5,6]; until the last century in Mexico and other countries, this was the accepted name for this disease. Suffering from nerves, in previous times, was described as a state of restlessness accompanied by difficulty falling asleep, lack of or increase in appetite, tachycardia, and despair. To this could be added skin problems, weaknesses, or insomnia [6]. Currently, the presence of an anxiety disorder can be referred to “el ansia” (nervousness or fear), which is another term used in traditional medicine that can be related to anxiety [7]. It was defined as physical discomfort, which manifested as restlessness and anxious breathing, and was caused by anguish or emotional distress. Mexico has wide plant diversity and a wealth of knowledge about using plants as remedies to treat different mental disorders, especially anxiety disorders. Furthermore, modern medicine recognizes these conditions as cultural filiation syndromes and recognizes their significance within specific cultural contexts [8].

*Argemone platyceras* Link & Otto is a plant native to Mexico and is located in the center of Mexico, from Veracruz to Puebla, Tlaxcala, and the State of Mexico. It lives in a temperate climate, between 1200 and 2400 m, and is associated with grasslands, oak, and pine forests. It is a small shrub that reaches a size of 30 cm to 1 m in height; it can be annual or can last more than two years. It also has a yellow latex. The leaves are whitish green, with indentations, and end in a thin spine. The flowers are white to slightly pale yellow and showy [9,10]. In popular medicine, it has been used when there is vaginal discharge to cure the kidneys (state of Hidalgo) and diabetes (Tlaxcala), and a maceration of the seeds with water is administered to remove waste from the eyes (Michoacán) [10]. The genus *Argemone* has been considered an important medicinal plant and has been employed as a remedy for different diseases, such as dysentery, asthma, and intestinal affections [11]. Chemical constituents and crude plant extracts have exhibited pharmacological activities like antibacterial [12,13], anti-HIV [14], anti-inflammatory [15], antistress, antiallergic [16], nematicidal [17], and antidiabetic activities [18,19]. This plant has been used ethnobotanically in the center of Mexico, especially in the Puebla and Tlaxcala (Mexico) regions, as a treatment for diabetes disease and to reduce the nerves (“los nervios”) and the anxiety (“la ansiedad”) caused by emotional distress.

Based on traditional medicine, this study aims to evaluate the chemical constituents of *A. platyceras* Link & Otto, a native species of Mexico, and its antidepressant- and anxiolytic-like effects on behavioral paradigms in mice.

## 2. Results

### 2.1. Isolation and Standardization of the Method of Extraction

Five hundred grams of grounded plant material was extracted twice with 500 mL of absolute MeOH at room temperature. After filtration, the solvent was evaporated to obtain the methanol extract. This procedure was completed twice for each plant material collected. The extract obtained was stored in the dark at 4 °C until later use. The yields are shown in Table 1.

To complete the extraction of the alkaloids, the MeOH Fc was dissolved in 200 mL of 0.5 N H_2_SO_4_ and kept in magnetic stirring for 24 h at rt. After this time, the solution was filtered through Whatman No. 1 paper and neutralized with NH_4_OH. Once the solution was neutralized, a chloroform extraction was performed to obtain the alkaloid extract (AlkExt), which was stored for analysis by mass spectrometry techniques and for pharmacological evaluation. The yields are shown in Table 2.

### 2.2. Chemical Composition by Mass Spectrometry Analysis

#### Direct Infusion Mass Spectrometry (DIESI) and UHPLC-ESI Analysis of AlkExt

**Figure 1:**
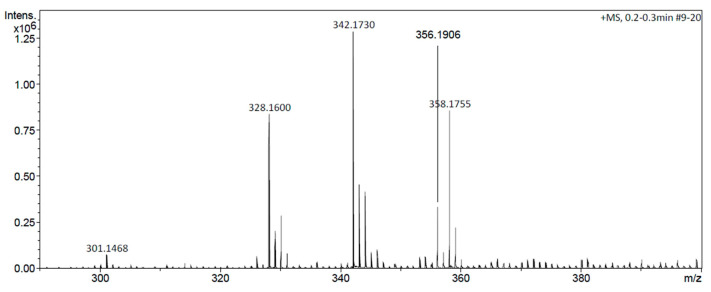
DIESI-MS spectra of the AlkExt of *A. platyceras* Link & Otto.

The AlkExt was first analyzed using DIESI-MS. As we can see in Figure 1, the highest peaks detected with molecular ions at *m*/*z* 328, 342, 356, and 358 were first assigned to two groups of alkaloids: berberin alkaloids (*m*/*z* 328, 342, and 358; scoulerine, tetrahydrocolumbamine and 8-(hydroxymethyl)-2,10-dimethoxy-6,8,13,13a-tetrahydro-5H-isoquinolino[2,1-b]isoquinoline-1,11-diol, respectively) and aporfine alkaloids (*m*/*z* 356; glaucine) [20]. In addition to these compounds, 2,3′,4,5′-Tetramethoxystilbene (*m*/*z* 301.14) was also detected.

**Figure 2:**
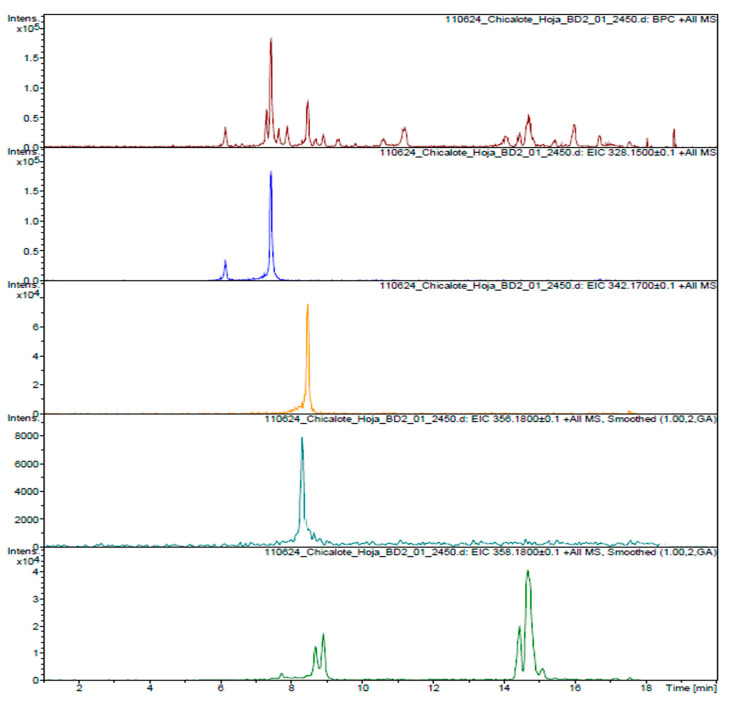
EIC chromatogram of the AlkExt of *A. platyceras* Link & Otto.

**Figure 3:**
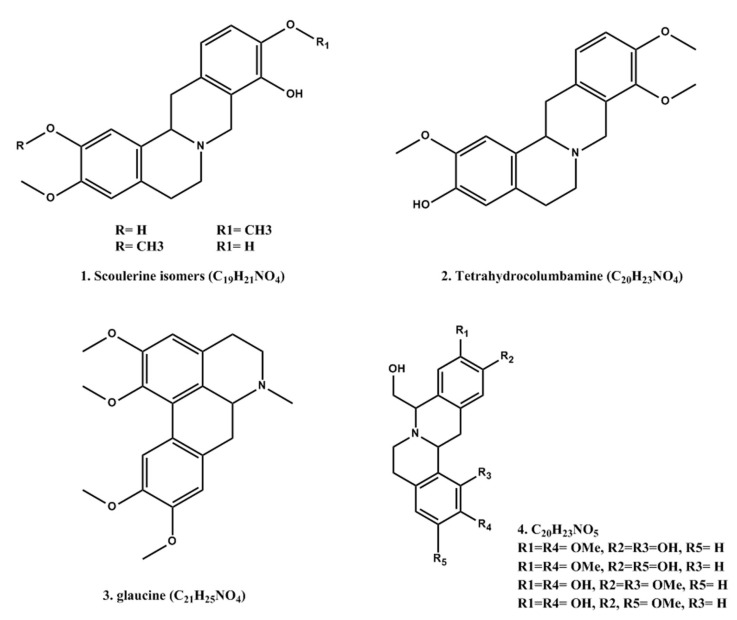
Alkaloid characterized in the AlkExt of *A. platyceras* from the EIC analysis.

To complete the structural characterization of the alkaloids, the AlkExt was subjected to a UPLC-MS analysis; according to Figure 2, different peaks were observed and analyzed by the extracted ion chromatogram (EIC) tool from the total ion chromatograph (TIC) to confirm the structural characterization of the compounds previously described. For the molecular ion at *m*/*z* 328.1600 [M + H]^+^, a molecular formula of C_19_H_20_NO_4_ (*m*/*z* 328.1543) was assigned to two peaks detected in the EIC analysis as isomers (Figure 2), and according to the ms/ms analysis, (*S*)-scoulerine (Figure 3) was assigned the main peak at rt 7.4 min. The peak at rt 6.1 min should be a scoulerine isomer; unfortunately, the low concentration made it difficult to complete the characterization. The next molecular ions at *m/z* 342.17 and 356.19 [M + H]^+^ were assigned to the molecular formulas C_20_H_23_NO_4_ and C_21_H_25_NO_4_ (*m/z* 342.1699 and 356.1856, respectively) (Figure 2 and Figure 3). According to the ms/ms analysis, two structures were confirmed for these formulas: (*S*)-tetrahydrocolumbamine and (*S*)-glaucine. Finally, for the molecular ion at *m/z* 358.17, at least four peaks were identified from the EIC analysis, and according to the ms/ms analysis, these compounds correspond to the (8S,13aS)-8-(hydroxymethyl)-2,10-dimethoxy-6,8,13,13a-tetrahydro-5H-isoquinolino[2,1-b]isoquinoline-1,11-diol isomers.

The ms/ms analysis showed the same fragmentation pattern, which indicates that there could be a variation in the methoxy and hydroxyl groups in the same aromatic ring without a change between both aromatic rings. Possible structures are proposed in Figure 3, and the relative percentages of the alkaloids detected in the chromatogram from the UPLC-EIC analysis of the AlkExt are shown in Table 3.

Once the AlkExt was standardized and characterized, pharmacological assays were performed to investigate its antidepressant- and anxiolytic-like effects.

### 2.3. Pharmacological Evaluation

Antidepressant-like effect

**Figure 4:**
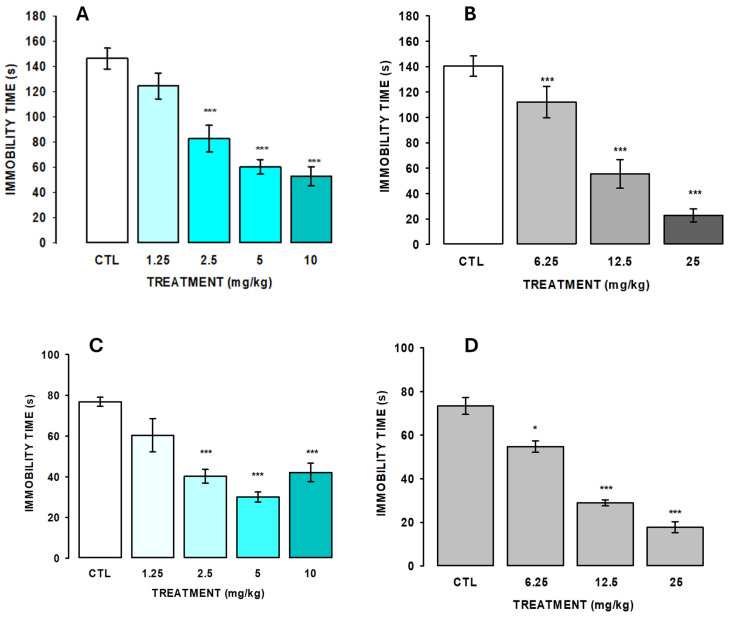
AlkExt at 1.25, 2.5, 5, and 10 mg/kg (**A**) and clomipramine at 6.25, 12.5, and 25 mg/kg (**B**) effects in the tail suspension test (TST). AlkExt at 1.25, 2.5, 5, and 10 mg/kg (**C**) and clomipramine at 6.25, 12.5, and 25 mg/kg (**D**) effects in the forced swimming test (FST). Bars represent the mean ± standard error of the mean of independent groups of 7 to 9 mice. CTL: administered group with saline solution (0.9% NaCl). Results analysis was performed using one-way analysis of variance (ANOVA) or Kruskal–Wallis one-way analysis of variance on ranks, followed by Holm–Sidak or Mann–Whitney U rank sum test pairwise multiple comparisons. *, *p* ≤ 0.05, ***, *p* ≤ 0.001.

Figure 4A shows the effect of AlkExt in the tail suspension test. The results showed that AlkExt caused a significant reduction in the immobility time at 2.5, 5.0, and 10 mg/kg in the control group (H = 26.515, fd = 4, *p* ≤ 0.001). However, no significant difference was observed between 5 and 10 mg/kg; this effect was similar to that caused by clomipramine (CIM; F_(3,28)_ = 246.4, <0.001).

As shown in Figure 4B, the acute oral administration of AlkExt reduced the immobility time at 2.5, 5.0, and 10.0 mg/kg (H = 23.5, fd = 4, *p* ≤ 0.001) in the control group (CTL), in a similar form to clomipramine (CIM; F_(3,28)_ = 85.365, *p* ≤ 0.001), and it reached the best effect at 5.0 mg/kg in the forced swimming test (FST). These results indicate that the acute oral administration of AlkExt caused an antidepressant-like effect without affecting the spontaneous ambulatory activity of the experimental subjects; as shown in Table 4, AlkExt at 1.25 to 10.0 mg/kg did not produce changes in the rearing number (F_(4,41)_ = 1.45, *p* = 0.23), nor did it modify the ambulatory activity (count number; F_(4,41)_ = 145, *p* = 0.23), which indicates that the significant reduction in the immobility behavior caused by AlkExt in both the TST and the FST is due specifically to an antidepressant-like effect.

The data show that AlkExt did not affect the spontaneous ambulatory activity at either of the doses that produced an immobility time reduction in the FST.

The data represent the mean ± standard error of the mean of the independent groups of 8 to 10 mice. Data analysis was performed using one-way analysis of variance (ANOVA).

Anxiolytic-like effect

The anxiolytic-like effect of AlkExt was explored in two predictive paradigms of anxiety that are widely recognized in the evaluation of antidepressant drugs and APIs: the EPM and HBT tests.

**Figure 5:**
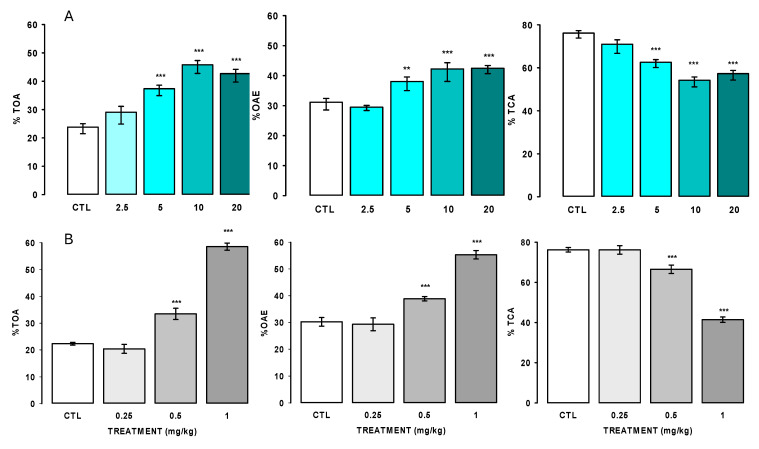
Anxiolytic-like effect of AlkExt at 2.5, 5, 10, and 20 mg/kg (**A**) and the diazepam at 0.25, 0.5, and 1 mg/kg (**B**) in the elevated plus maze test (EPM). TOA: percentage of time spent on the open arms; OAE: percentage of time on open arm entries; TCA: time spent on the closed arms expressed as percentage. CTL: control group, (0.9% NaCl solution), bars represent the mean ± standard error of the mean of independent groups of 8 mice. Results analysis was performed using the Kruskal–Wallis one-way analysis of variance on ranks and Tukey’s pairwise multiple comparison test **, *p* ≤ 0.01, ***, *p* ≤ 0.001.

As shown in Figure 5, our results showed that AlkExt significantly reduced the behaviors associated with high anxiety levels in both models. However, with diffident sensibility, in the EPM, AlkExt at 5, 10, and 20 mg/kg significantly increased both the frequency (F_(4,35)_ = 19.76, *p* < 0.001) and the time (F_(4,35)_ = 35.78, *p* < 0.001) spent on the open arms and caused a reduction in the time spent on the closed arms (F_(4,35)_ = 35.51, *p* < 0.001), while DZ was at 0.25 to 1.0 mg/kg (DZ %TOA F_(3,28)_ = 134.4; %OAE F_(3,28)_ = 50.90, *p* < 0.001; %TCA F_(3,28)_ = 90.55, *p* < 0.001). In the HBT, as Table 5 shows, oral treatment with AlkExt caused a clear anxiolytic-like effect from 2.5 to 20 mg/kg, increasing the exploratory behaviors, such as the frequency and the time spent exploring the holes, in the control group (F_(4, 35)_ = 14.9, *p* ≤ 0.001, F_(4,35)_ = 9.6, *p* ≤ 0.001, respectively); these are indices of low anxiety levels.

## 3. Discussion

Depression and anxiety are the more prevalent mood disorders. These disorders are highly comorbid and share several standard etiological processes. Therefore, treatments that prevent these illnesses could develop together. Despite the large arsenal of drugs available to treat these diseases, the development of new, effective, and safe drugs is necessary. Medicinal plants have shown their ability to alleviate depression and anxiety symptoms, especially the alkaloids from medicinal plants [21].

Different studies have demonstrated that *Argemone* sp. has diverse chemical constituents, and most of the isolated compounds belong to the class of alkaloids, in addition to terpenoids, flavonoids, phenolics, and other minor constituents of these plants. The main problem in isolating these compounds is the high amount of latex in the plant. In this regard, isolating compounds such as alkaloids is a substantial challenge. In this work, we improved the alkaloid extraction, and as shown in Figure 3 (Table 3), the main compounds obtained are alkaloids. The main compounds detected were 2,3′,4,5′-Tetramethoxystilbene (*m*/*z* 301.14, 3%), scoulerine (*m*/*z* 328.16, 19.8%), tetrahydrocolumbamine (*m*/*z* 342.17, 28.8%), 8-(hydroxymethyl)-2,10-dimethoxy-6,8,13,13a-tetrahydro-5H-isoquinolino[2,1-b]isoquinoline-1,11-diol (*m*/*z* 358.17, 22.8%), and glaucine (*m*/*z* 356.19, 11.1%).

The tail suspension test (TST) and the forced swim test (FST), the gold standards of the depression model, were used to evaluate AlkExt’s antidepressant-like effects. The TST and FST are widely used in the screening of antidepressant drugs due to their relative simplicity, accessibility, and reliability.

The FST involves placing an animal into a glass container of water, from which it cannot escape [22]. The rodent initially struggles to get out and eventually gives up and floats or moves to keep floating, i.e., it develops or adopts an immobility behavior. Although the behavioral repertoire of the animals in the FST is complex, the immobility time is considered a meaningful indicator of depression-like behavior. The TST is similar in some ways to the FST, but the tail suspends the animals so that they cannot escape or reach anything nearby. They struggle initially but eventually become immobile. In the TST, the mice remain suspended by their tails, eliciting active and passive behaviors to escape from this adverse condition [23]. This test is like the FST in that an animal’s immobility time is interpreted as depression-like behavior. In this test, mice that are administered antidepressant drugs before the test show more active escape responses than those without treatment [24]. Our results show that AlkExt in the TST caused a dose-dependent reduction in the passive immobility behavior from 2.5, 5, and 10 mg/kg doses and was significantly different from the saline-treated group. In the FST, it also caused a reduction in the immobility time, which was substantially different from the control group. Their best effect was found at 5 mg/kg, and it was no different from the 10 mg/kg dose. The AlkExt actions were similar to those produced for the clomipramine (CIM), in similar doses; CIM is a dibenzazepine-derivative antidepressant, which is an inhibitor of serotonin and norepinephrine reuptake. These results allow the exploration of the biochemical pathways involved in the extract’s antidepressant actions in the future. To avoid false positive results in the TST and the FST, such as a decrease in the immobility behavior due to unspecific side effects resulting from the ambulatory hyperactivity of experimental animals, caused by the possible psychostimulant effects of AlkExt [25], our study evaluated the spontaneous ambulatory activity of animals subjected to the TST and the FST in the OFT. Our results showed that the AlkExt did not affect locomotor activity at doses that produced their antidepressant-like effect. These results indicate that the extract actions in mice are due specifically to its antidepressant-like effect.

Anxiolytic-like effects of AlkExt

To evaluate anxiolytic-like effects, we used two behavioral paradigms that are widely recognized and validated for their sensibility in detecting anxiolytic-like effects regardless of the biochemical pathway underlying their anxiolytic actions: the HBT and the EPM. Our findings show that the oral administration of AlkExt caused a clear anxiolytic-like effect in two behavioral paradigms: the HBT and EPM; the anxiolytic actions of AlkExt were similar to those produced by diazepam, a benzodiazepine of clinical use [26]. In the HBT, AlkExt increased the time spent exploring the perforations. Although DZ increased exploratory behaviors at a more potent rate than AlkExt, the response profile in this paradigm was similar. Similarly, in the EPM, AlkExt caused a significant increase in exploratory behaviors, such as frequency (OAE) and time (TOA) spent on the open arms and a diminishing time spent on the closed arms. In this model, these behaviors indicate a diminishing of anxious behavior. So, our results show that AlkExt produced clear anxiolytic-like effects in the HBT and the EPM.

Finally, it is known that serotonin and dopamine play a pivotal role in the pathophysiology of depression and anxiety by modulating mood, reward, and stress response [27]. Previously, we described the antidepressant-like effects of an alkaloid aporphine-type extract in mice, and the neurochemical evaluation indicated that the extract at 10 mg/kg produced a generalized increase in the serotonin and dopamine turnover in the whole brains of mice, increasing the concentration of these neurotransmitters and their metabolism. Like CIM, these data suggest that the monoaminergic system participates in AlkExt antidepressant actions. However, more specific studies are necessary to determine the biochemical mechanisms underlying these actions [28,29].

The study of the Argemone species is necessary to indicate that the main limitation is the alkaloid extraction due to the high presence of latex in the plant, which could have an effect that masks some results. This study strongly suggests its therapeutic potential to treat the comorbidity of anxiety and depression disorders and supports further investigations in people with these diseases.

## 4. Materials and Methods

**Figure 6:**
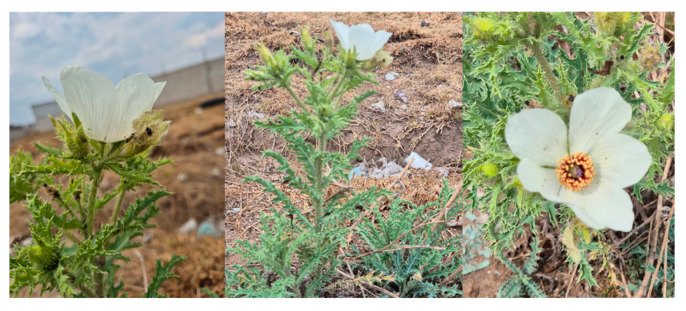
*Argemone platyceras* Link & Otto grows wild in the Apizaco region of Tlaxcala (México).

During the winter of 2023–2024, fresh plants of *A. platyceras* Link & Otto were collected at noon in the Apizaco, Tlaxcala (México) region (latitude 19.418, longitude −98.127) on the Apizaco-Puebla freeway near Tlaxcala City, Mexico (Figure 6). Plant identification was conducted by PhD Alfonso Daniel Gay González, and a specimen was deposited in the National Herbarium of Mexico (MEXU) (voucher number 645813). The specimens used for taxonomic identification were transported to the laboratory using the plant pressing technique. The ones used for the extraction were deposited in Ziploc bags to be transported, and the leaves and stems were dried at 25 °C for two weeks under dark conditions.

Afterwards, the plant material was milled to obtain 2–5 mm size particles that were extracted exhaustively at room temperature with methanol for 2 days. The methanol extract was then filtered through Whatman #1 paper and evaporated to dryness in a rotary evaporator under reduced pressure. The weights of the crude dried extracts were 131.5 g (18% yield) and 88.4 g (14.7% yield), respectively. The extracts were stored at room temperature and protected from light until their utilization.

### 4.1. Alkaloid Extraction and Quantitation [23]

The alkaloids were extracted from 100 mg of MeOH Fc dissolved in 200 mL of 0.5 N H_2_SO_4_ and kept in magnetic stirring for 24 h at rt. After this time, the solution was filtered through Whatman No. 1 paper and neutralized with NH_4_OH. Once the solution was neutralized, a chloroform extraction was performed to obtain the alkaloid extract (AlkExt), which was stored for analysis by mass spectrometry techniques and for pharmacological evaluation.

### 4.2. Analysis of Methanol Extract of A. platyceras Link & Otto [30]

Samples of the methanolic extract were dissolved in methanol and analyzed using ultra performance liquid chromatography–mass spectroscopy analysis (UPLC-ESI-MS). An Ultimate 3000 ultra-performance liquid chromatography (UPLC) system (Dionex corp., Waltham, MA, USA) with photodiode array detection (PAD) was coupled to a Bruker MicrOTOF-Q II system by an electrospray ionization (ESI) interface (Bruker Daltonics, Billerica, MA, USA); electrospray ionization (ESI) analysis was conducted using a Bruker micrOTOF-Q II. The compound related peaks were found in the positive and negative ion modes (ESI+). The capillary potential was −4.5 kV, with a dry gas temperature of 200 °C and a drying gas flow of 4 L/min. The total ion chromatograms were from *m*/*z* 100 to 1000. The obtained patterns were analyzed by a Bruker Compass Data Analysis 4.0 (Bruker Daltonics), which provided a list of elemental formulas using the molecular formula generator.

### 4.3. Animals

Adult male Swiss Webster mice (weighing 20–30 g) were obtained from the vivarium at the Instituto Nacional de Psiquiatría Ramón de la Fuente Muñiz (INPRFM) vivarium. All the animals were housed at eight per cage with free access to water and food in a temperature-controlled (21–22 °C) room under inverted light/dark conditions (12 h:12 h, lights on at 22:00 h). The animal experiments followed NIH guidelines for the care and use of laboratory animals, 8th edition (2011), and the Mexican legislation (NOM-062-ZOO-1999) of the Secretary of Agriculture, Livestock, Rural Development, Fisheries, and Food (SAGARPA). In November 2019, the Internal Committee for the Care and Use of Laboratory Animals (CICUAL) approved all the animal experimentation procedures (NC19054) used in this study. All the experiments were video recorded for later analysis.

Administration protocols.

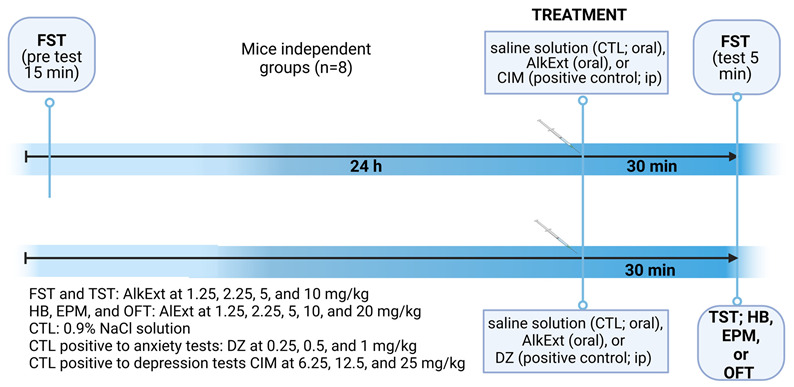



### 4.4. Evaluation of Depression-like Effects

The antidepressant-like actions of AlkExt were evaluated in the tail suspension (TST) and the forced swimming (FST) tests.

#### 4.4.1. Tail Suspension Test

In the TST, the mice are held individually by the tail in an acoustic and visually isolated wooden table 50 cm above a soft surface for 6 min, which elicits active and passive behaviors to escape from this adverse condition [24]. The long-lasting immobility was recorded during the final 4 min of the test. The mice received different treatments 30 min before the test. The immobility time was measured only when the mouse remained hanging wholly and passively motionless, and a reduction in the immobility time in the control group is considered an antidepressant-like effect. The test sessions were videotaped and later scored by two observers blinded to the treatments applied [23].

The acute effects of the AlkExt extract were evaluated in five independent groups of male mice who received an oral single administration of the AlkExt extract at 0, 1.25, 2.5, 5.0, and 10 mg/kg and were then evaluated 30 min later in the test.

#### 4.4.2. Forced Swimming Test

The mice were individually placed into glass cylinders (height: 21 cm, diameter; 14.5 cm) containing 15 cm of water at 23 ± 1 °C and forced to swim for a 15 min period (pre-test) to stimulate the immobility behavior, which was assessed in a second swim session (test session) 24 h later (5 min).

Drugs were administered 30 min before the test session. The test sessions were videotaped and registered by an observer who was unaware of the pharmacological treatments. Reduced accumulation of immobility time is interpreted as an antidepressant-like effect [28].

### 4.5. Open Field Test

In both the TST and FST, immobility time was measured, and antidepressants reduced this behavior. However, it is necessary to discard the idea that this diminishment is due to an increase in the spontaneous activity of experimental subjects due to drugs that can cause side effects. Hence, the ambulatory activity of animals subjected to the FST or TST is evaluated using the open field test. The OFT device comprises a polycarbonate box divided into six equal quadrants (40 × 30 × 20 cm). At the start of the test, the mouse is placed in the central part of the box. For five minutes, its ambulatory activity is measured as the number of times the animal stands up on its hind paws and crosses from one quadrant to another with all its body.

### 4.6. Anxiolytic-like Effects of AlkExt Were Evaluated in the Elevated Plus Maze (EPM) and Hole Board (HB) Tests

#### 4.6.1. Elevated Plus Maze Test (EPM)

The EPM is a widely validated model for measuring the anxiolytic-like effects of anxiolytic drugs, including active principles (APIs), in rodents [31].

The apparatus consists of two opposite open arms (30 × 8 cm), intersected (center platform) by two closed arms of the exact dimensions, with 19 cm high walls. The arms are connected to an 8 × 8 cm central square. The device is 55 cm above the floor in a dimly illuminated room. At the start of the test, the mouse is placed individually in the center of the plus maze, facing an open arm, for 5 min. It registers the number of entries (all paws on open or closed arms), the time spent on the open arms (TOA), and the time spent on the closed arms (TCA), which are expressed as a percentage.

% TOA = TOA/TCA × 100 (Time spent in Closed Arms; TCA), % Open Arm Entries (% OAE), OAE = Open Arm Entries/[Closed Arm Entries + Open Arm Entries] × 100.

Also, it measures the time on the central platform (TC) = [TOA + TBC] − 300; (300 s = test times, in such a way that TOA + TCA + TC = 300 s).

The percentage of time and the frequency of entries in the open arms are considered indices of the anxiety level [31].

#### 4.6.2. Hole Board Test

The hole board device consists of a 60 × 30 × 15 cm wooden box with four equidistant holes (1 cm diameter) on the floor. At the start of the test, the mouse is placed at the center of the box, and it registers the time and number of times that the mouse head-dips in one of the holes. It also measures the rearing number (when a mouse stands up on its hind legs) over a 5 min period. After each trial, the floor of the apparatus is carefully cleaned to remove traces of previous tests. A decrease in the time and number of head dips and the rearing number relative to the control group reveals a sedative action, while an increase in these same variables is considered an anxiolytic-like effect [30].

### 4.7. Data Analysis

The data were analyzed using either one-way analysis of variance (ANOVA) or the Kruskal–Wallis analysis of variance on ranks and the pairwise multiple comparisons using the Mann–Whitey rank sum test or Dunnet’s test when the analysis of variance showed a significant difference of *p* ≤ 0.05. All the statistical analyses were conducted using the Sigma Plot version 12.5 software, and the graphics were carried out using the Sigma Plot or Prisma version 9.0 software.

## 5. Conclusions

Traditional medicine needs to be supported by scientific studies to treat different diseases. In this study, a standardized method was used to obtain an extract mainly composed of 2,3′,4,5′-Tetramethoxystilbene (*m*/*z* 301.14, 3%), scoulerine (*m*/*z* 328.16, 19.8%), tetrahydrocolumbamine (*m*/*z* 342.17, 28.8%), 8-(hydroxymethyl)-2,10-dimethoxy-6,8,13,13a-tetrahydro-5H-isoquinolino[2,1-b]isoquinoline-1,11-diol (*m*/*z* 358.17, 22.8%), and glaucine (*m*/*z* 356.19, 11.1%). This extract (AlkExt) was pharmacologically tested, and the results indicate that a single oral administration with AlkExt caused robust anxiolytic- and antidepressant-like effects in mice without affecting the spontaneous ambulatory activity of the mice. The results strongly suggest its therapeutic potential to treat the comorbidity of anxiety and depression disorders. These results support the use of *A. platyceras* in traditional medicine in the treatment of anxiety disorders.

## Figures and Tables

**Table 1:** Yields of the fractions obtained from the *A. platyceras* methanol extraction.

Material Collected	MeOH Fc	Yield
1 December 2023 (500 g)	123.4 g	24.68% ^1^
2 February 2024 (500 g)	115.8 g	23.16% ^1^

^1^ Extraction yield is the average of two processes.

**Table 2:** Yields of the AlkExt obtained from the *A. platyceras* from methanolic extractions.

Material Collected	AlkExt	Yield
1 December 2023 (100 g)	2.4	2.4% ^1^
2 February 2024 (100 g)	3.1	3.1% ^1^

^1^ Extraction yield is the average of two processes.

**Table 3:** Retention time and relative percentage of the alkaloids detected in the chromatogram of the UPLC-EIC analysis of the AlkExt from *A. platyceras*.

Rt	Formula	[M + H]^+^ MW_detected_	[M − H]^+^ MW_exact_	% Relative
6.1	C_19_H_21_NO_4_	328.1577	328.1543	5.8
7.4	C_19_H_21_NO_4_	328.1570	328.1543	37.6
8.3	C_20_H_23_NO_5_	358.1693	358.1649	2.2
8.5	C_21_H_25_NO_4_	356.1894	356.1856	18.3
8.7	C_20_H_23_NO_4_	342.1725	342.1699	3.1
8.9	C_20_H_23_NO_5_	358.1581	358.1649	3.8
14.4	C_20_H_23_NO_5_	358.1668	358.1649	6.0
14.7	C_20_H_23_NO_5_	358.1664	358.1649	23.2

**Table 4:** AlkExt and clomipramine (CIM) effects on the locomotor activity of mice in the open field test (OFT).

Treatment/Doses (mg/kg)	Rearing Number	Count Number
CLT/0	29.2 ± 2.87	48.1 ± 3.15
AlkExt/1.25	34.0 ± 1.85	54.5 ± 4.54
AlkExt/2.25	33.25 ± 2.98	58.37 ± 3.42
AlkExt/5.0	32.7 ± 3.43	58.0 ± 5.39
AlkExt/10.0	38.75 ± 2.92	61.25 ± 3.39
	F_(4,41)_ = 1.45, *p* = 0.23	F_(4, 41)_ = 1.45, *p* = 0.23
CIM/CTL	42.00 + 2.98	52.12 + 3.28
CIM/6.25	33.25 + 189	52.87 + 3.30
CIM/12.5	34.37 + 2.06	55.75 + 4.42
CIM/25.0	35.12 + 2.74	50.50 + 4.14
	F_(3,28)_ = 2.57, *p* = 0.07	F_(3,28)_ = 0.33, *p* = 0.80

AlkExt; alkaloid extract, CIM; clomipramine. Rearing number: number of times the mouse stands on its hind legs. Count number: number of times the mouse crosses from one quadrant to another.

**Table 5:** Anxiolytic-like effect of AlkExt and diazepam (DZ) in the hole board test (HBT).

AlkExt	Rearing Number	Head-Dipping Time (s)	Head-Dipping Number
CTL	27.37 ± 2.7	21.68 ± 2.5	19.00 ± 1.7
2.5	32.12 ± 1.0	23.83 ± 2.0 ***	21.60 ± 1.5 **
5.0	32.75 ± 2.0	33.56 ± 1.7 **	27.75 ± 1.5 **
10.0	38.87 ± 1.7 ***	41.90 ± 1.8 ***	30.50 ± 1.5 ***
20.0	36.25 ± 2.1 ***	34.68 ± 2.3 ***	27.87 ± 1.3 **
	F_(4, 35)_ = 12.0, *p* ≤ 0.001	F_(4, 35)_ = 14.9, *p* ≤ 0.001	F_(4,35)_ = 9.6, *p* ≤ 0.001
DZ	Rearing number	Head-dipping time (s)	Head-dipping number
CTL	17.28 ± 0.47	22.15 ± 1.9	17.28 ± 0.4
0.25	19.28 ± 0.83	27.78 ± 3.16	19.28 ± 0.8
0.5	21.57 ± 0.68 ***	35.92 ± 1.8 ***	21.57 ± 0.6 *
1.0	26.00 ± 1.49 ***	63.24 ± 2.5 ***	26.0 ± 1.4 ***
	F_(3,24)_ = 15.43, *p* ≤ 0.001	F_(3,24)_ = 55.61, *p* ≤ 0.001	F_(3,24)_ = 15.43, *p* ≤ 0.001

Rearing number: number of times the mouse stands on its hind legs. Count number: number of times the mouse crosses from one quadrant to another. Head-dipping time: time that mouse spends head-dipping in one of the holes. Head-dipping number: number of times mouse dips head in one of the holes. Data represent the mean ± standard error of the mean of independent groups of 8 mice. Data analysis was performed using one-way analysis of variance (ANOVA), followed by Tukey’s pairwise multiple comparison test *, *p* ≤ 0.05, **, *p* ≤ 0.01, ***, *p* ≤ 0.001.

## Data Availability

All data are contained within the article.
